# The heterogeneity of reversion to normoglycemia according to prediabetes type is not explained by lifestyle factors

**DOI:** 10.1038/s41598-021-87838-z

**Published:** 2021-05-06

**Authors:** Carolina Giráldez-García, Lucía Cea-Soriano, Romana Albaladejo, Josep Franch-Nadal, Manel Mata-Cases, Javier Díez-Espino, Sara Artola, Rosario Serrano, Enrique Regidor, for the PREDAPS Study Group, Margarita Alonso, Beatriz Álvarez, Fernando Álvarez, J Carlos Álvarez, Mª del Mar Álvarez, J Joaquín Antón, Oriol Armengol, Luis Ávila, Carmen Babace, Lourdes Barutell, Mª Jesús Bedoya, Belén Benito, Beatriz Bilbeny, Marti Birules, Concepción Blanco, Mª Isabel Bobé, Carmen Boente, Antonia Borras, Remei Bosch, Mª Jesús Brito, Pilar Buil, J José Cabré, Ainoha Cambra, Francisco Carbonell, Francisco Carramiñana, Lourdes Carrillo, Ana Casorrán, Rafael Colas, Blanca Cordero, Xavier Cos, Gabriel Cuatrecasas, Cristina De Castro, Manuel De la Flor, Carlos De la Sen, Rosa Mar De Miguel, A María De Santiago, Mercedes Del Castillo, Mª Carmen Durán, Patxi Ezkurra, Paula Gabriel, Javier Gamarra, Francisco García, Luis García-Giralda, F Javier García-Soidán, Mª Teresa Gijón, Albert Goday, Ángel Gómez, María del Carmen Gómez, J Carles González, María González, Esteban Granero, Ángela Trinidad Gutiérrez, Félix Gutiérrez, Luisa Gutiérrez, M Ángel Gutiérrez, Ana Mª Hernández, Mercedes Ibáñez, Rosario Iglesias, Dimas Igual, Jaime Innenaraty, Yon Iriarte, Ángeles Jurado, Rafael Llanes, Flora López, Riánsares López, Ángela Lorenzo, Carmen Losada, Ramón Macía, Fernando Malo, José Mancera, Mª José Mansilla, Mª Teresa Marín, José Luis Martín, F Javier Martínez, Juan Martínez, Rosario Martínez, Mª Soledad Mayayo, J Javier Mediavilla, Luis Mendo, J Manuel Millaruelo, Alicia Monzón, Ana Moreno, Pedro Muñoz, Xavier Mundet, Teresa Mur, Emma Navarro, Jorge Navarro, Pedro Nogales, J Carlos Obaya, Francisco Javier Ortega, Francisca Paniagua, José Luis Pardo, Francisco Carlos Pérez, Pedro P Pérez, Neus Piulats, Raquel Plana, Nuria Porta, Santiago Poveda, Luis Prieto, Ramón Pujol, Jazmín Ripoll, Antonio Rodríguez, J José Rodríguez, Mª Angeles Rollán, Laura Romera, Jóse Félix Rubio, Antonio Ruiz, Irene Ruiz, Manuel Antonio Ruiz, Isabel Sáenz, Julio Sagredo, Alejandro Salanova, L Gabriel Sánchez, Manuel Sánchez, Gloria Sanz, Mateu Seguí, Dulce Suárez, Eduard Tarragó, Jesús Torrecilla, José Luis Torres, Merè Villaró, Carmen Yuste

**Affiliations:** 1 redGDPS Foundation, Madrid, Spain; 2 Del Tajo University Hospital, Madrid, Spain; 3 Department of Public Health and Maternal and Child Health, Faculty of Medicine, Complutense University of Madrid, Pza. Ramón y Cajal, S/N. Ciudad Universitaria, 28040 Madrid, Spain; 4 Barcelona City Research Support Unit/University Institute for Research in Primary Care Jordi Gol, Barcelona, Spain; 5 Biomedical Research Networking Centre Consortium on Diabetes and Associated Metabolic Disorders, Madrid, Spain; 6 Departament of Medicina, University of Barcelona, Barcelona, Spain; 7 La Mina Primary Care Center, Barcelona, Spain; 8 Tafalla Health Center, Navarra, Spain; 9 José Marvá Health Center, Madrid, Spain; 10 Martín de Vargas Health Center, Madrid, Spain; 11 Biomedical Research Networking Centre Consortium on Public Health and Epidemiology, Madrid, Spain; 12 Institute of Health Research in the Hospital Clínico San Carlos, Madrid, Spain; 13 CS De la Eria, Asturias, Spain; 14 CS Andrés Mellado, Madrid, Spain; 15 CS, La Calzada 2, Asturias, Spain; 16 CS Eras de Renueva, León, Spain; 17 CS Hereza, Madrid, Spain; 18 CS Murcia-Centro, Murcia, Spain; 19 EAP Poblenou, Barcelona, Spain; 20 Consultorio Almachar, Málaga, Spain; 21 CS Rodríguez Paterna, La Rioja, Spain; 24 EAP Raval Sud, Barcelona, Spain; 27 CS Sada, A Coruña, Spain; 28 EAP La Mina, Barcelona, Spain; 29 CS Porriño, Pontevedra, Spain; 30 CS Canal Salat, Baleares, Spain; 31 EAP Girona 2, Girona, Spain; 32 CS La Matanza, Baleares, Spain; 33 EAP Azpilagaña, Navarra, Spain; 34 EAP Reus-1, Tarragona, Spain; 35 CS Arrabal, Zaragoza, Spain; 36 CS Mislata, Valencia, Spain; 37 CS San Roque, Badajoz, Spain; 38 CS La Victoria de Acentejo, Santa Cruz de Tenerife, Spain; 39 CS Fuente de San Luis, Valencia, Spain; 40 CS Santoña, Cantabria, Spain; 41 CS Sta. María de Benquerencia, Toledo, Spain; 42 EAP Sant Martí de Provençals, Barcelona, Spain; 43 CAP de Sarrià, Barcelona, Spain; 45 CS Ntra. Sra. de Gracia, Sevilla, Spain; 46 Consultorio San Gabriel, Alicante, Spain; 47 EAP Pubillas Casas, Barcelona, Spain; 48 Unidad Docente de Atención Familiar y Comunitaria, Guadalajara, Spain; 50 CS Lavadores Vigo, Pontevedra, Spain; 51 CS Zumaia, Guipúzcoa, Spain; 52 EAP Badia del Vallès, Barcelona, Spain; 53 CS Medina del Campo Rural, Valladolid, Spain; 54 CS Don Benito Este, Badajoz, Spain; 55 CS Murcia Centro, Murcia, Spain; 57 CS Los Yébenes, Madrid, Spain; 58 Endocrinología Hospital del Mar, Barcelona, Spain; 59 CS Lasarte, Guipúzcoa, Spain; 60 CS Vélez-Málaga Norte, Málaga, Spain; 61 Girona 3, Girona, Spain; 62 CS Alcantarilla Sangonera, Murcia, Spain; 63 CS Vista Alegre Murcia, Murcia, Spain; 64 CS El Calero, Las Palmas, Spain; 65 CS Bombarda-Monsalud, Zaragoza, Spain; 66 CS Beraun, Guipúzcoa, Spain; 67 CS Ávila Sur Oeste, Ávila, Spain; 69 CS Vandel, Madrid, Spain; 70 CS Lain Entralgo, Madrid, Spain; 71 CS Manuel Encinas, Cáceres, Spain; 72 CS Hereza Leganes, Madrid, Spain; 73 CS Aizarnazabal-Getaria, Guipúzcua, Spain; 74 CS Salvador Caballero, Granada, Spain; 75 Villanueva de la Cañada, Madrid, Spain; 76 EAP Martorell, Barcelona, Spain; 77 CS Artilleros, Madrid, Spain; 78 CS Alcalá de Guadaira, Madrid, Spain; 79 UGC Adoratrices, Huelva, Spain; 80 CS Roces Montevil, Asturias, Spain; 81 CS Ares, A Coruña, Spain; 82 CS Ciudad Jardín, Málaga, Spain; 83 CS Martín de Vargas, Madrid, Spain; 84 CS General Ricardos, Madrid, Spain; 86 CS Federica Monseny, Madrid, Spain; 87 CS Yecla, Murcia, Spain; 88 CS Oñati, Guipúzcoa, Spain; 90 CS Burgos Rural, Burgos, Spain; 91 CS Cadreita, Navarra, Spain; 92 CS Torrero La Paz, Zaragoza, Spain; 93 CS Vecindario, Las Palmas, Spain; 94 CAP San Roque, Badajoz, Spain; 95 Unidad Docente de Medicina Familiar y Comunitaria, Cantabria, Spain; 96 EAP Carmel, Barcelona, Spain; 97 CAP Terrasa Sud, Barcelona, Spain; 98 CS Añaza, Santa Cruz de Tenerife, Spain; 99 CS Salvador Pau, Valencia, Spain; 100 CS Las Águilas, Madrid, Spain; 101 CS Chopera, Madrid, Spain; 102 CS Campos-Lampreana, Zamora, Spain; 104 CS Orihuela I, Alicante, Spain; 106 CS Mallen, Sevilla, Spain; 108 CS Ponteareas, Pontevedra, Spain; 109 CAP Terrassa Sud, Barcelona, Spain; 110 CS Jumilla, Murcia, Spain; 111 CS Cáceres-La Mejostilla, Cáceres, Spain; 112 EAP Tremp, Lleida, Spain; 114 EAP Anglès, Girona, Spain; 115 CS Villaviciosa de Odón, Madrid, Spain; 117 EAP Raval Nord, Barcelona, Spain; 118 CS Lasarte, Guipúzcua, Spain; 119 CS Pinto, Madrid, Spain; 120 EAP La Torrassa, Barcelona, Spain; 121 CS Agost, Alicante, Spain; 122 CS Espronceda, Madrid, Spain; 123 CS Los Rosales, Madrid, Spain; 125 CS Carballeda, Zamora, Spain; 127 CS San José centro, Zaragoza, Spain; 128 UBS Es Castell, Baleares, Spain; 130 EAP Bellvitge, Barcelona, Spain; 133 CAP Terrassa sud, Barcelona, Spain

**Keywords:** Epidemiology, Endocrine system and metabolic diseases

## Abstract

Healthy lifestyle interventions and drug therapies are proven to have a positive preventative influence on normal glucose regulation in prediabetes. However, little is known on the specific role that these factors play on reversion to normal glycemia according to type of prediabetes. We used data from the Observational prospective cohort study, The Cohort study in Primary Health Care on the Evolution of Patients with Prediabetes from 2012 to 2015. A total of 1184 individuals aged 30–74 years old were included and classified based on the ADA in three mutually exclusive groups using either fasting plasma glucose (FPG) levels (from 100 to 125 mg/dl, FPG group), HbA_1c_ (5.7–6.4%, HbA1c group) or both impaired parameters. Information on lifestyle factors and biochemical parameters were collected at baseline. Reversion to normal glucose regulation was calculated at third year of follow-up. Relationship of lifestyle factor and type of prediabetes with reversion were estimated using odds ratios (ORs) with 95% confidence intervals (95% CIs) adjusting by different groups of confounders. Proportion of reversion rates were 31% for FPG group, 31% for HbA1c group and 7.9% for both altered parameters group, respectively. Optimal life style factors such as BMI < 25 kg/m^2^[OR (95% CI): 1.90 (1.20–3.01)], high adherence to Mediterranean diet 1.78 (1.21–2.63) and absence of abdominal obesity 1.70 (1.19–2.43) were the strongest predictors for reversion to normal glucose. However, those did not modify the ORs of reversion to normal glucose. Taking as reference those with both impaired parameters, subjects with FPG impairment (FPG group) had an OR of 4.87 (3.10–7.65) and 3.72 (2.39–5.78) for HbA1c group. These estimates remained almost the same after further adjustment for biochemical parameters and lifestyle factors (4.55(2.84–7.28) and 3.09 (1.92–4.97), respectively). Optimal lifestyle factors showed to be a positive predictor for reversion to normal glucose regulation however, the differences of reversion risk according type of prediabetes are not explained by lifestyle factors.

## Introduction

The global prevalence of diabetes among adults aged 18 and beyond, has risen from 4.7% in 1980 to 8.5% in 2014, representing almost 422 million people by 2014^1^. In Spain the prevalence of diabetes in 2010 we 13.6%^2^. Moreover, the mortality of diabetes has scaled up to be positioned in the eight causes of dead rank worldwide^3^. Risk factors for developing diabetes type 2 have been well established and characterized^4–6^, which has allowed to implement interventive measures to reduce the burden of this disease across different healthcare systems. As an example, adherence to a healthy lifestyle [including healthy diet, smoking cessation, increase in physical activity, reduction in alcohol consumption, and reduction in body mass index (BMI)] at age 50–75, has been associated with six to ten years increase in life years and a significant improvement of quality of life. These implementations also resulted in a reduction in prevalence of major chronic diseases (including cancer, cardiovascular diseases and diabetes) compare to individuals with no vast improvement in lifestyle^7,8^.

Prediabetes status has been associated with a higher risk of developing diabetes type 2. This stage is characterized by either an impaired fasting plasma glucose (IFG) or impaired glucose tolerance (IGT), or elevated glycated hemoglobin A1c (HbA1c) depending on the established diagnostic criteria^9^. Prior research on this topic highlights how optimal lifestyle factors and drug therapies (majority oral antidiabetic medications) implementations are effective predictive positive factors to conversion to normal glucose regulation in subjects with prediabetes^5,10,11^. However, those interventions do not seem to play the same role depending on the type of prediabetes. There is sparsity of data on reversion to normal glucose according to IFG criteria, an interventional study of physical activity found higher reversion rates among individuals with IFG compared with those with IGT or both altered parameters^12^. In addition, a recent study observed how individuals with HbA1c levels in range of prediabetes were less likely to revert to normal glycaemia after physical activity recommendations^12^. Although these interventions could be effective, some authors have evaluated the role of phenotypic and genetic variables to determine the effectiveness of a lifestyle intervention to prevent diabetes^13^.

This apparent heterogeneous association, between optimal lifestyles and reversion to normal glycaemia regulation according prediabetes type, could be the cornerstone for individualized prevention strategies in subjects with prediabetes. However, there is little evidence on how healthy lifestyle factors can explain the variation in the proportion of patients reverting to normal glucose regulation according to type of prediabetes. In order to develop a better understanding of the following matter, the current study aimed to study the heterogeneity in the reversal rate to normal glucose regulation among the three groups and to study the key role of lifestyle factors in predicting the reversal using a prospective cohort of individuals with prediabetes followed up by primary care physicians in Spain.

## Material and methods

### Study design

The Cohort study in Primary Health Care on the Evolution of Patients with Prediabetes (PREDAPS Study) is a prospective study encompassing two cohorts of patients: those with prediabetes status and those with normal glycemia (i.e. non-prediabetes neither diabetes) with the attempt to study the progression, prognosis and behavior of prediabetes. Details on information and design published previously by the same authors^14^. Briefly, this prospective study conducted at the primary care setting, started in 2012. To be member of the prediabetes cohort individuals aged 30–74 years old were included when meeting the following prediabetes criteria based on the definition established by American Diabetes Association^15^ using only FPG and HbA1c parameters as there were not data available to identify impaired glucose tolerance (IGT). First group, namely (1) group 1 (isolated IFG), included all individuals with FPG 100–125 mg/dl, (2) group 2 (isolated A1c group), included all individuals with HbA1c 39–47 mmol/mol (5.7%–6.4%) and (3) group 3 (both altered parameters group), included all individuals with HbA1c 39–47 mmol/mol (5.7–6.4%) and FPG 100–125 mg/dl. Participants aged 30–74 years old with HbA1c < 39 mmol/mol (< 5.7%) and FPG < 100 mg/dl were assigned to the normoglycemia cohort. Individuals with the following criteria were excluded (to be members) from the study cohort: diabetes, terminal disease, pregnancy, surgery, or hospital admission in the previous 3 months at study entry, or any hematologic disease, which could alter HbA1c values. A total of 2022 individuals gave their written informed consent for participation: 1184 subjects with prediabetes and 838 without impaired glucose metabolism.

**Figure 1:**
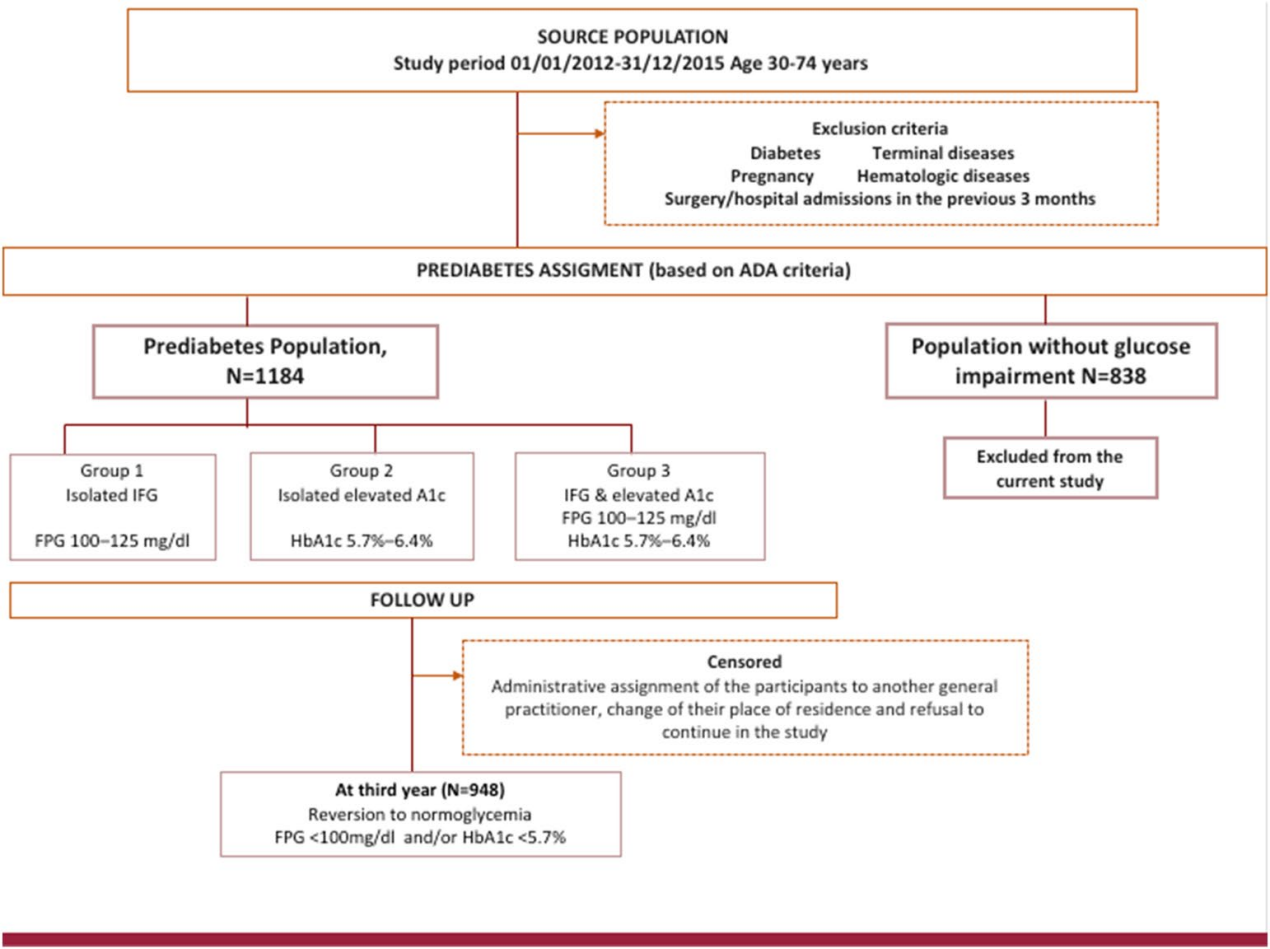
Proportion of patients reverting from prediabetes to normal glycaemia stratified by type of prediabetes.

The present study analyzed the relationship between lifestyle and other variables (i.e. including lifestyle factors and metabolic conditions) measured at baseline and the situation of reversion to normal glycaemia in the third year of follow-up among the cohort of subjects with prediabetes. Thus, out of 1184 subjects with prediabetes, a total of 948 (80.1%) attended their third follow up visit and were retained to be members of the final cohort, therefore remaining patients were excluded. The main causes of exclusion were the administrative assignment of the participants to another general practitioner, change of their place of residence and refusal to continue in the study. Reversion to normal glucose regulation, was considered if FPG and HbA1c values were FPG < 100 mg /dl and HbA1c < 39 mmol/mol (< 5.7%), respectively, at third year of follow up (Fig. 1).

### Data collected

The study protocol has been published^14^. Basically, the questionnaire included more than 200 items and all physicians were trained to carry out the interview, collect information and complete the questionnaire. During the medical visit blood and urine analyses were requested to determine FPG, HbA1c, lipid profile, transaminases, blood count, iron levels and renal function. Of note, all variables were treated as categorical data. Similarly, visit a physical examination was performed, which included anthropometry and determination of blood pressure. Three readings of height, weight, waist circumference, systolic and diastolic blood pressure were taken. The mean of the three readings was used for the analysis. The questionnaire of health surveys carried out at national level was used to measure smoking and physical activity. Smoking habit was classified into three mutually exclusive categories: current smoker, former smoker, and non- smoker. Individuals were asked to state which of the following alternatives best reflected their alcohol consumption frequency: never drinker, former drinker, occasional drinker, or daily drinker. For the present analysis individuals were classified into non-drinkers, occasional drinkers and daily drinkers. Physical activity data were collected based on the frequency—number of times in the last two weeks—and amount—mean time in minutes for each session as well as different types of physical activity, and, on the basis of the data collected, the minutes per week of physical activity performed by each participant were estimated. Subjects were classified into two categories to their compliance with the World Health Organization (WHO) physical activity recommendations—accumulate at least 150 min per week of moderate aerobic activity or 75 min per week of vigorous aerobic activity—, or an equivalent combination of moderate and vigorous physical activity^16^

Diet information was obtained by a simplified 20-item food frequency questionnaire, based on validated instrument which included standard portions of foods^17,18^. And several response categories: daily consumption, ≥ 3 times / week, 1–2 times / week, < 1 time / week, never or almost never. The foods were: dairy products, meats, cold meats and sausages, fish, eggs, legumes, potatoes, vegetables, fruit, rice and pasta, bread, cakes or sweets, olive oil, other oils, animal fat, fried foods, ready meals, preserved food, nuts, bag snacks. Adherence to the Mediterranean diet was estimated through an adaptation of the score used by *Panagiotakos* in the ATTICA study^19^. A score of 0 was considered minimum adherence, compared to 80, which would be maximum adherence. Adherence to the Mediterranean diet was grouped into three categories low (0–53 points), medium (54–59 points) and high (60–80 points).

Overweight and general obesity was defined as a Body Mass Index (BMI) ≥ 25 g/m^2^, and abdominal obesity as a waist circumference ≥ 102 cm in men and ≥ 88 cm in women. Hypertension was defined as systolic blood pressure ≥ 140 mmHg, or diastolic blood pressure ≥ 90 mmHg, or current use of antihypertensive treatment or having a personal history of hypertension. Hypercholesterolemia was defined as total serum cholesterol ≥ 250 mg/dl, high-density cholesterol level (HDL-C) of as < 40 mg/dl in men and < 50 mg/dl in women, and hypertriglyceridemia as serum level of triglycerides ≥ 200 mg/dl.

### Statistical analysis

A descriptive analysis of the distribution of demographic characteristics, lifestyle variables, obesity, and hypertension and biochemical parameters, according to type of prediabetes was performed using the chi-square test, Pearson’s chi square (categorical variables), *p* values < 0.05 were considered statistically significant. Then it was calculated the percentage of subjects who reverted to normal glycaemia according to these variables and types of prediabetes. Multivariate logistic regression was used to estimate odds ratios (OR) with 95% confidence intervals (CIs) to quantify the association between demographic characteristics, lifestyle variables, obesity, hypertension and biochemical parameters with reversion to normal glycaemia after adjustment for age and sex. ORs of reversion to normal glycaemia associated with type of prediabetes was estimated using three sequential models of adjustment: adjusting for age and sex (Model A); hypertension, hypercholesterolemia, HDL levels and triglycerides (Model B), and alcohol consumption, smoking, BMI, abdominal obesity, physical activity, adherence to Mediterranean diet (Model C). Confounders were included in each model based on its significant association (*p* < 0.05) or based on established knowledge of acting a risk factor biological mechanism of action (i.e. BMI). Each successive model included the factors from the previous model. Finally, prediabetes was further subdivided using as cut-off levels FPG < 110 and ≥ 110 mg/dl and HbA1c < 42 and ≥ 42 mmol/mol (< 6 and ≥ 6%), respectively, and the relationship between subtype of prediabetes and the reversion to normal glycaemia was also estimated by models A, B y C. Statistical analyses were performed using the STATA package version 12.0 (StataCorp LP, College Station, TX, USA).

### Ethics approval

The study was classified by the Spanish Drug and Health Product Agency as a Non-Interventional (Observational) Post-Authorization Study, and the protocol was approved by the Parc de Salut Mar Clinical Research Ethics Committee in Barcelona. Informed consent was obtained from all participants and/or their legal guardians. Authors confirm that all methods were performed in accordance with the relevant guidelines and regulations.

## Results

### Baseline characteristics

Among our cohort of prediabetes, mean age was 58.7 years (median: 60 years). Amongst them, 21% of patients were classified as having isolated impaired FPG (group 1), 27.6% had isolated elevated HbA1c levels (group 2) and 50.9% had both altered parameters (group 3). Table1 shows the baseline characteristics of study cohort according to type of prediabetes. There was an inverse proportion of men and women according to each prediabetes criteria; a total of 61% of group 1 population were men, corresponding percentages were 38.9% for group 2 and 50.3% for group 3, respectively (*p* < 0.001). Distribution of age was similar within groups, although group 1 tended to be younger. In terms of alcohol consumption, the proportion of daily drinkers were 29% for group 3 compared to 19.5% for group 2 and 34% for group 1. Besides group 3 presented a higher proportion of BMI > 25 kg/m^2^ and abdominal obesity. There were no differences in distribution of remaining lifestyle factors as smoking, physical activity and adherence to diet. Those in group 3 had a higher frequency of hypertension and triglycerides levels and there were no differences in distribution of hypercholesterolemia or HDL levels.

**Table 1:** Baseline characteristics of study cohort individuals according type of prediabetes.

Characteristics	Group 1* Isolated impaired FPG		Group 2^ϕ^ Isolated elevated HbA1c		Group 3^ψ^ Both altered parameters		*p* value Group 1 versus group 2	*p* value Group 1 versus group 3	*p* value Group 2 versus group 3
	N	%	N	N	N	%			
**Sex**							0.003	0.010	< 0.001
Women	79	38.9	160	61.1	240	49.7			
Men	124	61.1	102	38.9	243	50.3			
**Age**							0.129	< 0.001	0.181
30–49 years	46	22.7	43	16.4	60	12.4			
50–64 years	104	51.2	137	52.3	241	49.9			
65 + years	53	26.1	82	31.3	182	37.7			
**Smoking**							0.403	0.050	0.032
Current smoker	28	13.8	52	19.8	77	15.9			
Former smoker	96	47.3	94	35.9	180	37.3			
Never smoker	79	38.9	116	44.3	229	46.8			
**Alcohol consumption**							0.018	0.121	0.001
Daily drinker	69	34	51	19.5	140	29			
Ocassionally drinker	84	41.4	116	44.3	187	38.7			
Never drinker	50	24.6	95	36.3	156	32.3			
**BMI**							< 0.001	< 0.001	0.516
Overweight/Obese (> 25 kg/m^2^)	169	83.3	212	80.9	448	83.3			
Normal weight (up to 25 kg/m^2^)	34	16.7	50	19.1	35	16.7			
**Obesity abdominal**							< 0.001	< 0.001	0.536
Waist ≥ 88/102 cm	119	58.6	161	61.5	362	74.9			
Waist < 88/102 cm	84	41.4	101	38.5	121	25.1			
**Physical activity**							0.275	0.443	0.833
Do not follow OMS recommendations	112	55.2	146	56.2	112	52.0			
Follow OMS recommendations	91	44.8	114	43.8	232	48			
**Adherence to Mediterranean diet**							0.031	0.931	0.006
Low	51	25.1	92	35.1	125	25.9			
Medium	98	48.3	98	37.4	236	48.9			
High	54	26.6	72	27.5	122	25.2			
**Hypertension**							< 0.001	0.007	0.161
Yes	130	64.0	151	57.6	359	74.3			
No	73	36.0	111	42.4	124	25.7			
**Hypercholesterolemia**							0.379	0.199	0.061
Yes	112	55.2	167	63.7	292	60.5			
No	91	44.8	95	36.3	191	39.5			
**Low HDL levels mg/dL**							0.084	0.044	0.684
Yes	38	18.7	53	20.2	125	25.9			
No	165	81.3	209	79.8	358	74.1			
**Hypertriglyceridemia mg/dl**							0.002	0.008	0.872
Yes	47	23.2	59	22.5	161	33.3			
No	156	76.8	203	77.5	322	66.7			

*Chi square of heterogeneity.

*Group 1, Isolated impaired FPG. Defined as FPG: 100–125 mg/dl and HbA1c: < 39 mmol/mol (< 5.7%).

^ϕ^Group 2 Isolated elevated HbA1c. Defined as FPG: < 100 mg/dl and HbA1c:39–47 mmol/mol (5.7–6.4%).

^ψ^Group 3 Both altered parameters. Defined as FPG: 100–125 mg/dl and HbA1c:39–47 mmol/mol (5.7–6.4%).

### Reversion rates according to type of prediabetes

**Figure 2:**
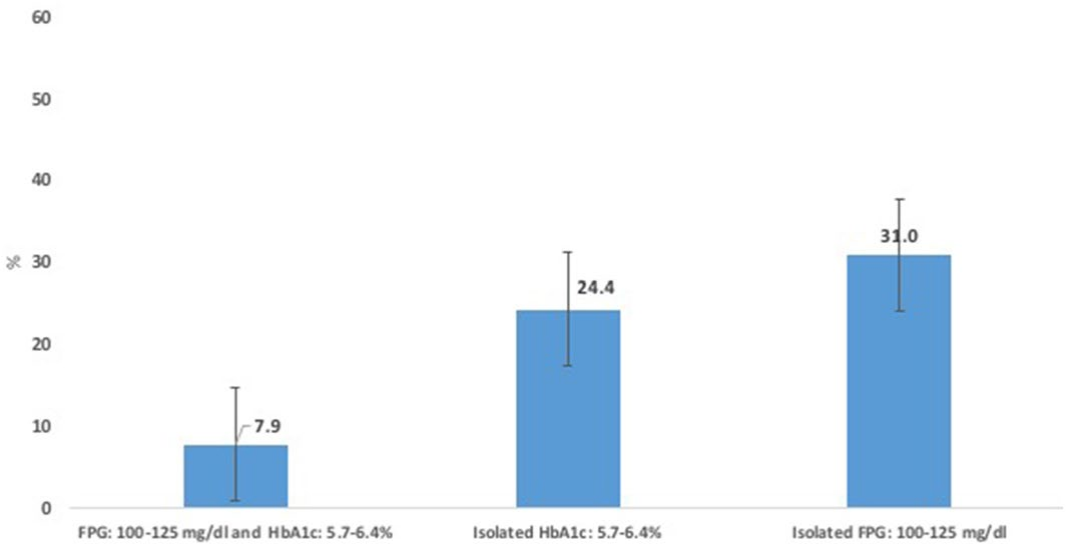
Proportion of patients reverting from prediabetes to normal glycaemia stratified by type of prediabetes.

**Figure 3:**
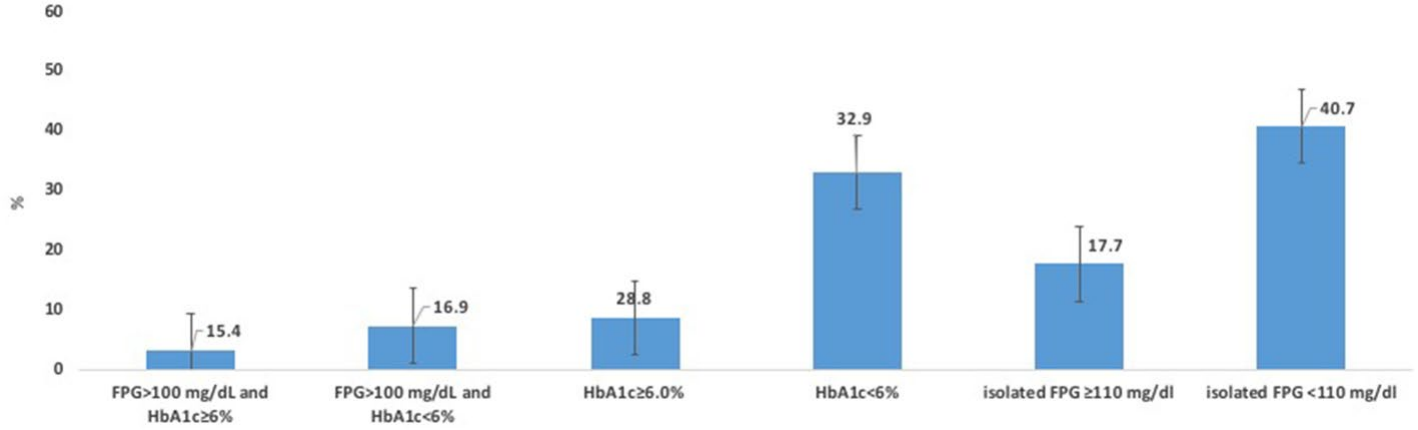
Proportion of patients reverting from prediabetes to normal glycaemia stratified by subtype of prediabetes.

At third year of follow up, there were a total of 165 (17.4%) patients who reverted to normal glucose regulation. When stratifying by type of prediabetes, a total of 7.9% of subjects in group 3 reverted to normal glycaemia, being the lowest proportion compared with 31% for group 1 and 24.4% for group 2, respectively (Fig. 2). Additionally, we subdivided the diagnostic criteria of prediabetes using as cut-off levels FPG < 110 and > 110 mg/dl and HbA1c < 42 and ≥ 42 mmol/mol (< 6 and ≥ 6%), respectively. Individuals with HbA1c levels ≥ 42 mmol/mol (< 6%) had the lowest reversion rates (3.2% for those with FPG: 100–125 mg/dl and 8.7% with FPG < 100 mg/dl), while those with isolated FPG < 110 mg/dl and isolated HbA1c < 42 mmol/mol (< 6%) obtained the highest reversion rates (40.7% and 32.9%) (Fig. 3).

### Factors associated with the reversion to normoglycemia

Table 2 shows the percentage of reversion according to each baseline characteristic factor as well as the OR of reversion. We did not observe any association with sex. There was a trend towards a decreased likelihood of reversion with the increase in age. Lifestyle factors such as BMI < 25 kg/m^2^ [OR 1.90 (95% CI 1.20–3.01)] compared to BMI ≥ 25, absence of abdominal obesity [OR 1.70 (95% CI 1.19–2.43)] compared of having a waist circumference ≥ 102 cm in men and ≥ 88 cm in women, a high adherence to Mediterranean diet [OR 1.78 (95% CI 1.21–2.63)] compared to having low/median adherence and following the WHO recommendations on physical activity [OR 1.48 (95% CI 1.04–2.10)] compared to not following them showed to be positive predictive factors associated with reversion to normal glycaemia. Not having hypertension shown to be associated with reversion to normoglycaemia. There was no association with biochemical parameters such as hypercholesterolemia, HDL low levels or hypertriglyceridemia.

**Table 2:** Percentage of reversion to normal glucose regulation and odds ratio (OR) according to the characteristics of the subjects.

Characteristics, N = 948	Percentage of reversion	Odds ratio (95% confidence interval)*
**Sex**		
Women	16.7	1.00
Men	18.1	1.06 (0.75–1.49)
**Age**		
30–49 years	31.5	2.92 (1.82–4.69)
50–64 years	15.6	1.17 (0.78–1.76)
65 + years	13.6	1.00
**Smoking**		
Current smoker	17.2	1.00
Former smoker	17.8	1.23 (0.74–2.04)
Never smoker	17.1	1.19 (0.71–1.99)
**Alcohol consumption**		
Daily drinker	14.6	1.00
Occasionally drinker	19.9	1.33 (0.85–2.07)
Never drinker	16.6	1.10 (0.66–1.82)
**BMI**		
Overweight/Obese (> 25 kg/m^2^)	16.2	1.00
Normal weight (up to 25 kg/m^2^)	26.1	1. 90 (1.20–3.01)
**Physical activity**		
Do not follow OMS recommendations	14.9	1.00
Follow OMS recommendations	19.4	1.48 (1.04–2.10)
**Adherence to Mediterranean diet**		
Low/medium	17.9	1.00
High	17.2	1.78 (1.21–2.63)
**Obesity abdominal**		
Waist ≥ 88/102 cm	14.8	1.00
Waist < 88/102 cm	22.9	1.70 (1.19–2.43)
**Hypertension**		
Yes	14.7	1.00
No	23.1	1.53 (1.06–2.19)
**Hypercholesterolemia (mg/dl)**		
Yes	17.3	1.00
No	17.5	1.02 (0.72–1.45)
**Low HDL levels (mg/dl)**		
Yes	18.5	1.00
No	17.1	0.98 (0.65–1.47)
**Hypertriglyceridemia (mg/dl)**		
Yes	14.6	1.00
No	18.5	1.38 (0.93–2.05)

*Sex and age adjusted odds ratio, except the odds ratios according sex and age.

All variables were considered and treated as categorical variables.

### Role of Lifestyle factors on reversion according to type of prediabetes

Using as reference group 3 (i.e. participants with both glycemic parameters altered) when adjusting by age and sex, the OR of reversion of prediabetes was 4.87 (95% CI 3.10–7.65) for group 1 and 3.72 (95% CI 2.39–5.78) for group 2. When adding biochemical parameters as well as hypertension (Model B), OR remained almost constant: 4.78 (95% CI 3.03–7.55) and 3.59 (95% CI 2.30–5.60), respectively. Finally, when including lifestyle factors (Model C), OR did remain almost the same: 4.52 (95% CI 2.84–7.18) and 3.43 (95% CI 2.17–5.42) (Table 3). Also, when subdividing prediabetes cohort according to levels of FPG and HbA1c, the OR in de Model C which was similar to OR in the Model B. Taking as reference those with HbA1c levels ≥ 42 mmol/mol (≥ 6%) and FPG 100–125 mg/dl, the OR for reversion after adjusting for all factors (Model C) were as follows: isolated FPG < 110 mg/dl: 18.21 (95% CI 8.08–41.06), isolated FPG ≥ 110 mg/dl: 5.75 (95% CI 2.30–14.37), isolated HbA1c < 42 mmol/mol (< 6%): 13.34 (95% CI 6.03–29.52), isolated HbA1c ≥ 42 mmol/mol (≥ 6%): 2.70 (95% CI 0.97–7.51), and HbA1c < 42 mmol/mol (< 6%) and FPG 100–125 mg/dl: 4.36 (95% CI 1.94–9.80).

**Table 3:** Reversion to normal glucose regulation. Odds ratio (and 95% confidence interval) according prediabetes type and according prediabetes subtype.

	Model A	Model B	Model C	Model D
**Prediabetes type**				
Group 3, Both altered parameters	1.00	1.00	1.00	1.00
Group 2, Isolated elevated HbA1c	3.72 (2.39–5.78)	3.59 (2.30–5.60)	3.43 (2.17–5.42)	3.09 (1.92–4.97)
Group1, Isolated FPG	4.87 (3.10–7.65)	4.78 (3.03–7.55)	4.52 (2.84–7.18)	4.55(2.84–7.28)
**Prediabetes subtype**				
HbA1C ≥ 42 mmol/mol(≥ 6%) and FPG 100–125 mg/dl	1.00	1.00	1.00	1.00
HbA1c < 42 mmol/mol (< 6%) and FPG 100–125 mg/dl	4.54 (2.03–10.17)	4.46 (1.99–9.99)	4.36 (1.94–9.80)	4.47 (1.98–10.06)
Isolated HbA1c ≥ 42 mmol/mol (≥ 6%)	2.81 (1.02–7.74)	2.75 (0.99–7.61)	2.70 (0.97–7.51)	2.43 (0.84–7.05)
Isolated HbA1c < 42 mmol/mol (< 6%)	14.65 (6.73–31.91)	14.11 (6.44–30.94)	13.34 (6.03–29.52)	11.84 (5.30–26.46)
Isolated FPG ≥ 110 mg/dl	6.18 (2.50–15.30)	6.03 (2.43–14.98)	5.75 (2.30–14.37)	5.99 (2.38–15.13)
Isolated FPG < 110 mg/dl	19.76 (8.84–44.15)	19.28 (8.60–43.25)	18.21 (8.08–41.06)	18.47 (8.14–41.90)

Model A: Adjusted by sex and age.

Model B: Model A plus hypertension, hypercholesterolemia, HDL levels and Triglycerides.

Model C: Model B plus alcohol consumption, smoking, BMI, abdominal obesity, physical activity, adherence to Mediterranean diet.

All variables were considered and treated as categorical variable.

Model D: Model A plus alcohol consumption, smoking, BMI, abdominal obesity, physical activity, adherence to Mediterranean diet plus hypertension and all the ratio.

## Discussion

### Main findings

Reversion to normal glucose regulation at the third year of follow-up was almost four times higher in subjects with isolated impaired HbA1c and almost five times higher in subjects with isolated impaired FPG, compared with subjects with both altered parameters. Adjustment for lifestyles did not modify markedly the magnitude of association between type of prediabetes and reversion to normoglycemia. Thus, the differences found across groups cannot be explained via lifestyle factors.

### Comparison with existing literature

Previous studies evaluating the proportion of reversion to normoglycaemia were heterogeneous in design, duration of follow-up and criteria definition, yielding a broad range of reversion rates^20–24^. Only few did it according to prediabetes criteria^13,25^. A Japanese study similar to ours observed a greater proportion of reversion rates among those with elevated HbA1c levels^25^. Our results showed an opposite trend resulting in lower rates for individuals with HbA1c levels ≥ 6.0% regardless FPG levels, similar to the results provided by a British study^13^. Using the same prediabetes criteria than ours, other study observed that individuals who reverted to normoglycaemia had a low insulin resistance and optimal beta-cell function at baseline^26^. Therefore, it would be reasonable to think that individuals with HbA1c ≥ 6% at baseline, had an increased insulin resistance and/or a decreased beta cell function, which might explain the lowest reversion rates found. Although we did not capture fasting plasma insulin, we used other parameters that might serve as a proxy for recognizing insulin resistance. We calculated the triglyceride (TG) glucose (TyG) index and several lipid ratios: TG/HDL ratio, the total TC/HDL ratio, and the LDL/HDL ratio and analysed them in tertile strata. Individuals with HbA1c ≥ 6% at baseline presented higher levels (Tertile 1) compared with individuals with only impaired FPG. For example, almost 50% of subjects with only impaired FPG were located in tertile 3 while 38.9% of only impaired HbA1c were in tertile 1 and 33% for those with both altered parameters (*data not shown*).

Baseline characteristics such as age less than 50 years, normal weight, absence of abdominal obesity, physical activity, adherence to Mediterranean diet and absence of hypertension have been associated with a higher likelihood to normal glucose regulation. There are studies in subjects with prediabetes that evaluate reversion to normoglycemia through intervention trials focusing on optimal lifestyle actions. The vast majority, although not all^13,27^, draw similar conclusions than ours^4,20,21,23,24,28^. Several trials that compared diet and physical activity promotion programs versus usual care in person with prediabetes reported reversion to normoglycemia between 20 and 52%^29^. Those findings have been confirmed in an intervention trial among persons with high cardiovascular risk^30^.

Both, obesity and body fat distribution are critical factors to decrease insulin sensitivity and B cells function^31^. Physical activity causes increased glucose uptake into active muscles balanced by hepatic glucose production and it improves insulin action^32^. There is also evidence that Mediterranean diet improves insulin sensitivity and prevents from diabetes^33^. In our study, at baseline, only BMI and waist circumference showed an association with FPG, HbA1c, triglycerides and cholesterol, but not the rest of lifestyles (Supplementary Table 1). However, baseline characteristics such as normal weight, absence of abdominal obesity, physical activity, adherence to Mediterranean diet shown association with a higher likelihood to normal glucose regulation. It is probable that individuals reverting to normoglycemia followed an optimal lifestyle behaviour long time before baseline state.

### Strengths and limitations

This is the first study evaluating the role of optimal lifestyle factors in the reversion to normoglycemia according type of prediabetes. Our study highlights the feasibility of conducting a prospective study, with data collected nation-wide by primary care physicians during routine clinical practice. Analytical determinations were performed at different laboratories. This fact could result in some source of misclassification. Since each subject was assigned to the same laboratory during the follow-up, this limitation should be minor and non-differential in relation to the outcome, as it is unlikely the relation between the methods employed by specific laboratories and reversion.

A potential source of misclassification when classifying subjects according to FPG levels cannot be ruled out. HbA1c reflects average plasma glucose over the previous eight to 12 weeks^34^, while FPG is subjected to daily variation levels^35^. If any substantial impact of misclassification, we would not be able to find the important observed differences to reversion according isolated impaired FPG levels. In addition, this study did not consider participants with prediabetes diagnosed based on oral glucose overload^14^, therefore it was not possible to estimate the prognosis for different categories according to this criterion. Although this is a limitation, in any case its impact would be minor as this measure is rarely used in routine practice.

Although 24-h dietary recall and dietary records have been used to measure usual dietary intake, both instruments are expensive and unrepresentative of usual intake and therefore, inadequate for the assessment of past dietary intake^36,37^. The food frequency questionnaire is the most commonly used instrument to assess past dietary intake in epidemiological studies. While the questionnaire of other Spanish study (PREDIMED study) used 14 item food, the PREDAPS Study used 20 in order to include some foods that are eaten as substitutes for the foods that are part of the Mediterranean diet. Therefore, the subjects with high adherence to the Mediterranean diet in the present analysis are strict adherents to this diet, since in the calculation of the score the absence of consumption of these substitute foods has been weighted more. Perhaps this explains that in these subjects the reversion to normoglycemia is 78% higher than in the rest of the subjects. On the other hand, an overestimation of physical activity cannot be excluded due to a possible recall bias of the activities carried out in the last two weeks. However, it is unlikely that this overestimation is differential with respect to the type of prediabetes of the subjects.

Researchers were unable to determine a time-dependent variable. However, the vast majority of the factors considered in the present study are chronic conditions or long-term lifestyle factors not susceptible to a fast variation within the follow-up during the study period. Likewise, we did not include in the analyses the existence or not of pharmacological treatment, as antihypertensive agents and lipid lowering drugs. In any case, there no was association between pharmacological treatment and reversion to normal glycemia. The odds ratio of association was 0.90 (0.62–1.29) in the case of use of antihypertensive agents and 1.06 (0.72–1.55) in the case of lipid lowering drugs*.*

Finally, since reversion to normoglycemia might not be a permanent condition (i.e. some subjects might change from first visit to third) we decided to use as a cutoff point the third visit of follow up to ensure a minimum time-lapse to measure the reversion. The subjects who did not reach the third visit were excluded. When evaluating the baseline characteristics of the dropped-out subjects, there were no significant substantial differences with respect to the subjects who remained in the study (Supplement Table 2). Therefore, a possible selection bias should not be ruled out, it should be minor.

Because the group sizes are small, the confidence intervals of the estimates in the different prediabetes categories overlap. However, in the analyzed prediabetes categories, the heterogeneous distribution of the estimates conforms to what is known about the FPG and/or HbA1c values used to consider prediabetes. Therefore, the magnitude of the association found could be very useful for decision-making in clinical practice.

### Implications for research and/or practice

The criteria of prediabetes are a controversial topic^38,39^. On the other hand, our findings suggest that beyond optimal lifestyles, FPG and HbA1c could be in themselves key markers to revert to normal glycaemia in subjects with prediabetes. Since 41% and 33% of the subjects with isolated FPG < 110 mg/dl and isolated HbA1c < 6% reverted to normoglycemia, respectively, this might lead into an overdiagnosis of prediabetes. Perhaps those subjects would not be the specific target for intensification of optimizing lifestyle factors and other actions such as initiating antidiabetic therapy. Further studies evaluating the role of optimal lifestyles in prognostic results, among subjects with prediabetes classified with different criteria, will be very useful to harmonize definitions on prediabetes and to better identify specific subjects with a low and high probability of normalizing glycaemia levels.

## Conclusions

In conclusion, optimal lifestyle factors showed to be a positive factor to reversion to normoglycaemia after three years of follow up in our prediabetes cohort however, they do not seem to explain differences in the reversion to normal glucose regulation according type of prediabetes.

## Supplementary Information

Supplementary Information 1.

Supplementary Information 2.

## Data Availability

Data will be available upon request.

## Abbreviations

ADA: American Diabetes Association
BMI: Body mass index
CI: Confidence intervals
FPG: Fasting plasma glucose
HbA1c: Glycated haemoglobin A1c
HDL-C: High-density cholesterol level
IFG: Impaired fasting plasma glucose
IGT: Impaired glucose tolerance
OR: Odds ratios
WHO: World Health Organization
