# Supplementary material for: The heterogeneity of reversion to normoglycemia according to prediabetes type is not explained by lifestyle factors
Source: Sci Rep. 2021 May 6;11:9667. doi: 10.1038/s41598-021-87838-z (PMC8102601; doi:10.1038/s41598-021-87838-z)
Supplement: Supplementary file 2 — Supplementary Information 2. [file 41598_2021_87838_MOESM2_ESM.docx]

**The heterogeneity of reversion to normoglycemia according to prediabetes type is not explained by lifestyle factors.**

Carolina Giráldez-García1,2, Lucía Cea-Soriano3, Romana Albadalejo3, Josep Franch-Nadal1,4,5,6, Manel Mata-Cases1,7, Javier Díez-Espino1,8, Sara Artola1,9, Rosario Serrano1,10, Enrique Regidor1,3,11,12 for the PREDAPS Study Group

(1) redGDPS Foundation, Madrid

(2) Del Tajo University Hospital, Madrid

(3) Department of Public Health and Maternal and Child Health, Faculty of Medicine, Complutense University of Madrid, Madrid

(4) Barcelona City Research Support Unit / University Institute for Research in Primary Care Jordi Gol, Barcelona

(5) Biomedical Research Networking Centre consortium on Diabetes and Associated Metabolic Disorders, Madrid

(6) Departament of Medicina. University of Barcelona, Barcelona

(7) La Mina Primary Care Center, Barcelona

(8) Tafalla Health Center, Navarra

(9) José Marvá Health Center, Madrid

(10) Martín de Vargas Health Center, Madrid

(11) Biomedical Research Networking Centre consortium on Public Health and Epidemiology, Madrid

(12) Institute of Health Research in the Hospital Clínico San Carlos, Madrid

Corresponding author:

Dra. Lucía Cea Soriano,

Departamento de Salud Pública y Materno Infantil, Facultad de Medicina.

Universidad Complutense de Madrid

Pza. Ramón y Cajal, s/n. Ciudad Universitaria.

28040 Madrid, Spain

E-mail: luciaceife@gmail.com

ORCID: 0000-0002-7051-0730

Phone: +34 (91) 394 20 45

**Table 1** Baseline characteristics of study cohort individuals among those who dropped out from the study and those included.

| **Characteristics** | **Patients dropped out from the study** | | **Patients included in the study** | | ***p* value*** |  |
| --- | --- | --- | --- | --- | --- | --- |
|  | N | % | N | % |  | |
| **Sex** |  |  |  |  | 0.281 | |
| Women | 110 | 46.6 | 479 | 50.5 |  | |
| Men | 126 | 53.4 | 469 | 49.5 |  | |
| **Age** |  |  |  |  | 0.272 | |
| 30-49 years | 38 | 16.1 | 149 | 15.7 |  | |
| 50-64 years | 107 | 45.3 | 482 | 50.8 |  | |
| 65+ years | 91 | 38.6 | 317 | 33.4 |  | |
| **Smoking** |  |  |  |  | 0.686 | |
| Current smoker | 40 | 16.9 | 157 | 16.6 |  | |
| Former smoker | 85 | 36.0 | 370 | 39.0 |  | |
| Never smoker | 111 | 47.0 | 421 | 44.4 |  | |
| **Alcohol consumption** |  |  |  |  | 0.368 | |
| Daily drinker | 58 | 24.6 | 260 | 27.4 |  | |
| Occasionally drinker | 92 | 39.0 | 387 | 40.8 |  | |
| Never drinker | 86 | 36.4 | 301 | 31.8 |  | |
| **BMI** |  |  |  |  | 0.271 | |
| Overweight/Obese (>25 kg/m^2^) | 200 | 84.7 | 829 | 87.4 |  | |
| Normal weight (up to 25 kg/m^2^) | 36 | 15.3 | 119 | 12.6 |  | |
| **Obesity abdominal** |  |  |  |  | 0.275 | |
| Waist ≥ 88/102 cm | 92 | 39.0 | 437 | 46.2 |  | |
| Waist <88/102 cm | 144 | 61.0 | 509 | 53.8 |  | |
| **Physical Activity** |  |  |  |  | **0.046** | |
| Do not follow OMS recommendations | 92 | 39.0 | 437 | 46.2 |  | |
| Follow OMS recommendations | 144 | 61.0 | 509 | 53.8 |  | |
| **Adherence to Mediterranean diet** |  |  |  |  | 0.278 | |
| Low/Medium | 166 | 70.3 | 700 | 73.8 |  | |
| High | 70 | 29.7 | 248 | 26.2 |  | |
| **Hypertension** |  |  |  |  | 0.433 | |
| Yes | 153 | 64.8 | 640 | 67.5 |  | |
| No | 83 | 35.2 | 308 | 32.5 |  | |
| **Hypercholesterolemia** |  |  |  |  | 0.150 | |
| Yes | 130 | 55.1 | 571 | 60.2 |  | |
| No | 106 | 44.9 | 377 | 39.8 |  | |
| **Low HDL levels** |  |  |  |  | **0.027** | |
| Yes | 70 | 29.7 | 216 | 22.8 |  | |
| No | 166 | 70.3 | 732 | 77.2 |  | |
| **Hypertriglyceridemia** |  |  |  |  | 0.849 | |
| Yes | 65 | 27.5 | 267 | 28.2 |  | |
| No | 171 | 72.5 | 681 | 71.8 |  | |

*Chi square of heterogeneity
